# A reinforcement-learning approach to efficient communication

**DOI:** 10.1371/journal.pone.0234894

**Published:** 2020-07-15

**Authors:** Mikael Kågebäck, Emil Carlsson, Devdatt Dubhashi, Asad Sayeed

**Affiliations:** 1 Department of Computer Science and Engineering, Chalmers University of Technology, Gothenburg, Sweden; 2 Department of Philosophy, Linguistics, and Theory of Science, University of Gothenburg, Gothenburg, Sweden; Royal Holloway University of London, UNITED KINGDOM

## Abstract

We present a multi-agent computational approach to partitioning semantic spaces using reinforcement-learning (RL). Two agents communicate using a finite linguistic vocabulary in order to convey a concept. This is tested in the color domain, and a natural reinforcement learning mechanism is shown to converge to a scheme that achieves a near-optimal trade-off of simplicity versus communication efficiency. Results are presented both on the communication efficiency as well as on analyses of the resulting partitions of the color space. The effect of varying environmental factors such as noise is also studied. These results suggest that RL offers a powerful and flexible computational framework that can contribute to the development of communication schemes for color names that are near-optimal in an information-theoretic sense and may shape color-naming systems across languages. Our approach is not specific to color and can be used to explore cross-language variation in other semantic domains.

## Introduction

The study of word meanings across languages has traditionally served as an arena for exploring which categorical groupings of fine grained meanings tend to recur across languages, and which do not, and for deriving on that basis a set of generalizations governing cross-language semantic variation in a given domain.

There is a long history of proposals that attempt to characterize how humans manage the effort of communication and understanding [1] and how this management can be affected by environmental demands [2]. One such increasingly influential proposal is that language is shaped by the need for *efficient communication* [3–7], which by its nature involves a trade-off [8, 9] between simplicity, which minimizes cognitive load, and informativeness which maximizes communication effectiveness. Specifically, they propose that good systems of categories have a near-optimal trade-off between these constraints. This trade-off is couched in the classic setting of Shannon information theory [10] which considers the fundamental laws of transmitting information over a noisy channel. Examples formalized in information-theoretic terms include suggestions that word frequency distributions, syllable durations, word lengths, syntactic structures, and case marking all facilitate efficient communication (see [3, 4] and references cited therein). The information theoretic view leads naturally to view the symbolic linguistic terms used for the communication as *codes* that create *partitions* of semantic spaces.

Given the principle of efficient communication, a fundamental challenge is to seek a concrete computational mechanism that could lead to optimal or near optimal communication schemes. Here we propose *Reinforcement learning* (RL) as a potential computational mechanism that may contribute to the development of efficient communication systems. Various systems, both artificial and in nature, can be represented in terms of the way they learn environmental interaction strategies that are near-optimal using RL techniques that employ reward/punishment schemas [11–13]. RL’s basis in operations research and mathematical psychology and ability to provide quantitative and qualitative models means it can be applied to a wide range of areas [13].

RL appears to be transparently implemented in neural mechanisms, for example, in dopamine neuron activity. For this reason, RL is increasingly recognized as having scientific value beyond mere computational modeling of decision-making processes [13–15]. That RL appears to be biologically so well-embedded implies that it can be seen as a general cognitive mechanism and used in an immediate way to make hypotheses about and interpretations of a variety of data collected in behavioral and neuroscientific studies.

**Fig 1:**
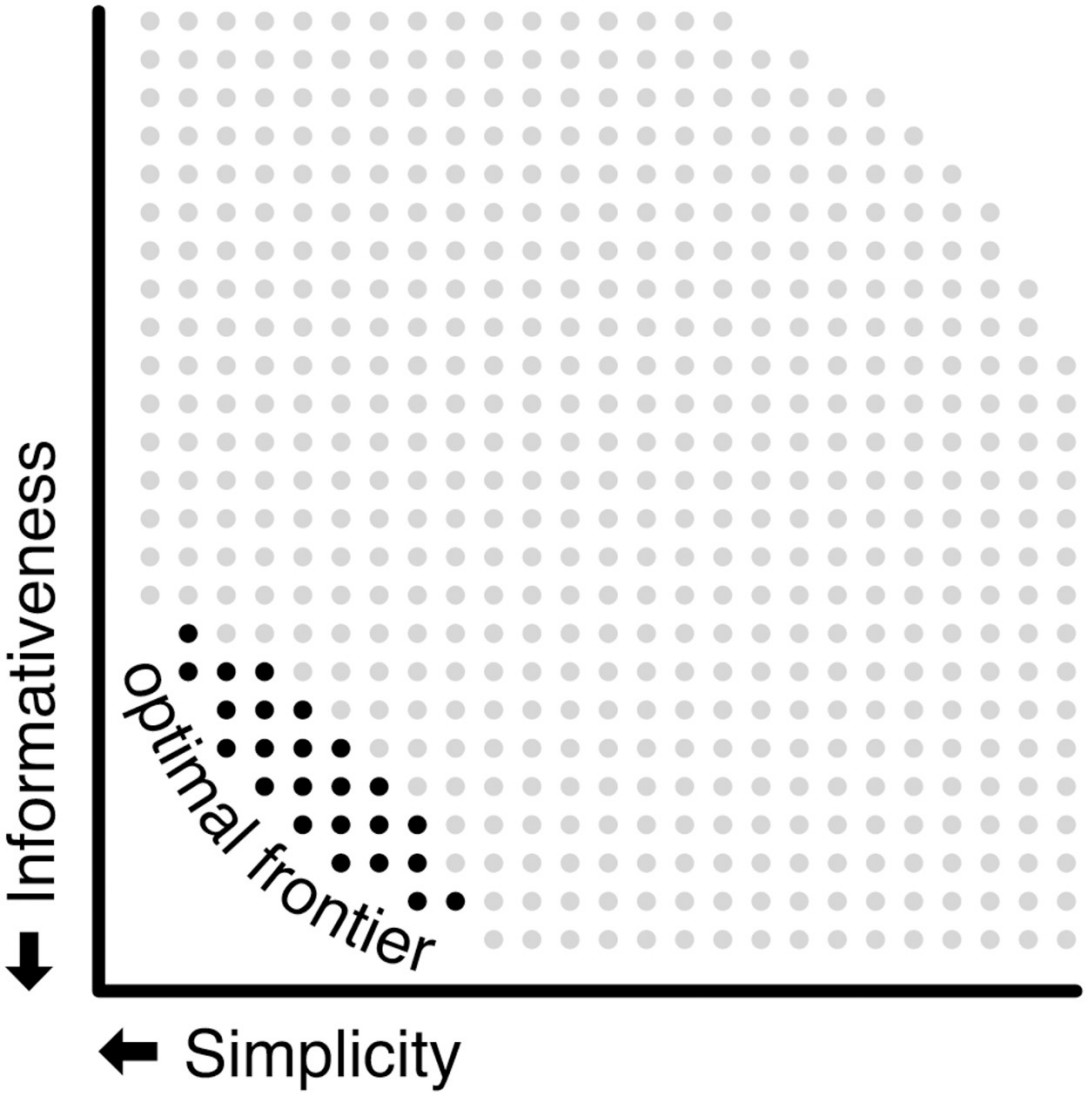
Human semantic systems will tend to lie along this optimal frontier of achievable efficiency in communication systems. Reprinted with permission from [21].

The availability of a growing suite of environments (from simulated robots to Atari games), toolkits, and sites for comparing and reproducing results about RL algorithms applied to a variety of tasks [16–20] makes it possible to study cognitive science questions through a different lens using RL. Cognitive science experiments are often carried out in real life settings involving questionnaires and surveys that are costly and sometimes suffer from high variability in responses. If simple RL algorithms are indeed a good proxy for actual human learning, then insights about questions of universals in language learning could be obtained very cheaply and reliably via controlled experiments in such *in silico* settings. Our approach could be used to explore various trade-offs at the heart of efficient communication [3]. Some languages are *simple* i.e. have few color terms while others have more color terms and are hence more *informative*. There is a tradeoff between these two properties and our framework can be used to test the prediction that human semantic systems will tend to lie along or near the optimal frontier of the region of achievable efficiency in communication systems as depicted schematically in Fig 1, see also [3, 21] for more discussion on this. Representing the question as an accuracy vs. complexity tradeoff specific to the domain of color terms, Zaslavsky et al. [22] demonstrate that a number of human languages, English included, come very close to that frontier. As pointed out by a referee, it is interesting to compare the approach here to Zaslavksy et al [22] who derive efficient naming systems as solutions to a differential equation implied by an information bottleneck (IB) loss function with terms to maximize information transfer and minimize lexicon complexity (which works out to, essentially, lexicon size). In contrast, this work considers a setting where two RL agents communicate through a noisy channel about color tiles, also observed through a noisy channel, and have to eventually agree on a communication protocol. The RL agents’ reward function is based on a similarity function in CIELAB space. We show that the resulting communication protocols satisfy the same efficiency measures that were used to define the information bottleneck, although the system was not explicitly optimized for these quantities. The environmental and communication noise rate ends up playing a similar role to the complexity penalty in the IB formulation (although with different dynamics over time), by reducing lexicon size. Thus, the two approaches are complementary: while the IB principle offers a descriptive analysis and establishes fundamental information–theoretic limits on the efficiency and complexity of communication schemes our approach is an algorithmic prescriptive route to how such optimal or near optimal schemes could be obtained.

While there may be reason to think that RL has a deep biological basis, in this work, we do not focus on the specifics of the underlying neurocognitive mechanism. Rather we demonstrate that very simple RL mechanisms are able to generate partitions for efficient (and near optimal) communication. We demonstrate this with a focus on questions about the universality of color categories and words in language. While there has been previous work [23] on computational mechanisms involving naming games for the emergence of universality of color names, our work is the first to provide a mechanism based on a fundamental machine learning paradigm (reinforcement learning) that is also biologically plausible.

### Linguistic background on color identification

#### A theory of universals

Color naming universals have a long history in linguistic research [24]. At an individual level, color perception is subjective; it differs for biological reasons across individuals (extreme examples being colorblindness and tetrachromacy). There are commonly-observed differences in individual color-naming choices. What is “turquoise” to one person may be a variant of “blue” to another. Nevertheless, within the same linguistic milieu, there is overall agreement as to color-naming; most English-speaking people recognize the typical supermarket tomato as “red”.

Berlin and Kay showed across a survey of 20 languages that there are strong consistencies in color naming and produced a set of universals: e.g., there are a maximum of eleven major color categories and, where fewer than eleven are realized for a given language, there is a standard pattern of emergence. This work came under methodological criticism [25, 26], particularly the use of standardized color systems to abstract away from the interactional and cultural basis of color identification.

Given this methodological conflict, is it really the case that such universals are artifacts of methods of investigation that take color communication out of its natural context in human linguistic interaction? Accounting for patterns of wide but constrained variation that have been observed empirically is a central challenge in understanding why languages have the particular forms they do.

Color terms represent a limited semantic domain with easily manipulated parameters. By gradual changes of color value, an experimenter can manipulate red into orange, unlike other semantic domains, where the distinctions between potential referents (e.g., “car” vs. “truck”) are not easily captured in explicit terms. In addition, recent work [4, 5] argues that color categories in language should support efficient communication.

#### Color naming models

**Fig 2:**
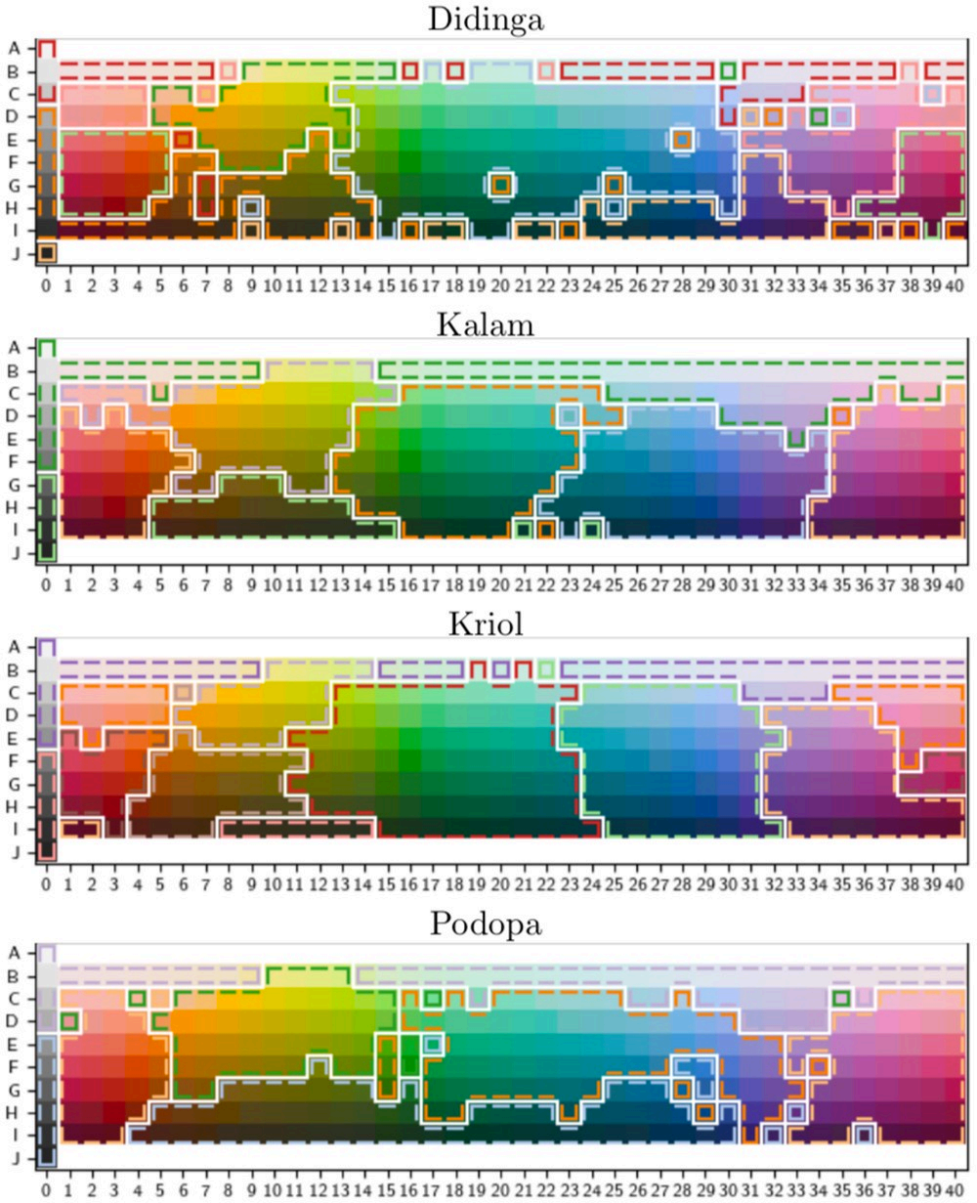
Reproduced color mode maps [4] corresponding to four randomly selected languages of the WCS.

Developed in 1905, the Munsell color system uses three color dimensions (hue, value, and chroma) to represent colors based on an “equidistance” metric calibrated experimentally by Albert Munsell. The World Color Survey (WCS; e.g. Fig 2) uses the Munsell color system in a matrix arranged by 40 hues, 8 values (lightness), and at maximum chroma (saturation). A color map can be developed for a particular language by asking speakers of that language to name each color. Color identification boundaries can be compared across languages using the WCS mapping.

The WCS color map technique enables the testing of automatic systems to partition colors. Regier et al. [27] experiment with partitioning the color space using a distance metric as a clustering criterion. They find a good distance metric by translating the WCS color map to the CIELAB space. CIELAB enables the translation of the WCS colors to a three-dimensional space, wherein the WCS colors appear to take an irregular spherical form. Regier et al. then use a standard “well–formedness” metric, which is essentially placing similar colors together and dissimilar apart. (Technically, this is called correlation clustering, which we explain later in the paper.) This allows them to automatically construct color partitions in the CIELAB space. Regier et al. find correspondences between optimal color partitions and observed color maps from human surveys as well as determine that rotating the WCS color space for a given observed color map causes reduced well–formedness in the corresponding CIELAB space. This is preliminary evidence for the optimality of color spaces in human language in relation to a well–formedness trade-off statistic.

Following their earlier work, Regier et al. adopt an information-theoretic approach [4] by introducing a communication system between two agents for multiple semantic domains (including color) and the corresponding notion of reconstruction error as the relative entropy (Kullback-Leibler divergence). The relative entropy is computed between the speaker’s model and the listener’s model of the probability that a particular term encodes a particular color. This becomes the communication cost of a color labeling system. They then show that real-world color-naming systems not only tend to have high well–formedness, but they also have low communication cost. A similar framework is adopted by Gibson et al. [5].

### Approach and contributions

This work focuses specifically on the role of speaker-listener communication efficiency in the partitioning of color spaces. To this end, we set up a two-agent paradigm closely mirroring the information-theoretic frameworks [3–5] that represent a series of negotiations between speaker and listener in the context of a “game”. Agent-based simulations are widely used in the study of the development of communication systems, including color communication [23, 28–30]. The basic paradigm used in our work is one in which the speaker and listener both begin with a set of available words (represented as integer identifiers) associated with a map of color “tiles”, where regions on the map are represented by the words. However, the speaker and the listener have different randomly-initialized maps. The speaker agent chooses a color tile and sends the listener agent the word that represents the region in which the tile is located. The listener agent then selects a tile that is in the region from its own map that most likely to be represented by that word in the speaker map. A reinforcement learning paradigm is used, as above, to update the parameters representing the shape of the maps, so that the game is run over many iterations.

This approach is a highly constrained representation of the “real-world” scenario of many speakers negotiating meaning in a speech community. Constrained simulations of communicative phenomena can allow the identification of plausible hypotheses about the factors that affect the corresponding real-world scenario, assuming that at least part of the expected behavior is reflected in the simulation.

In this work, we find that our two-agent simulation closely tracks the behavior of the languages in the World Color Survey in terms of both communication efficiency and perceptual well–formedness, relative to the number of primary color terms used. These are clearly separable from a random baseline and an idealized color map based on the CIELAB color space. Furthermore, the similarity of the color maps derived from the two-agent setting to the WCS maps remains relatively stable as the number of words are varied. We vary other metrics, such as perceptual and communication noise, to make predictions about color term convergence and demonstrate the flexibility of the model. The naturalness and stability of the model are evidence that our agent simulation paradigm is a suitable setting for investigation and hypothesis generation about cognitive and environmental effects on color communication in linguistic settings.

Enabled by recent advances in deep reinforcement learning [16, 18–20], this work therefore makes a methodological contribution to the study of the development of meaning in human languages given communicative factors. Our approach can offer complementary insight to the recent approach of Zaslavsky et al. [22] who argued that languages efficiently compress ideas into words by optimizing the *information bottleneck* (IB) trade-off between the complexity and accuracy of the lexicon.

## Efficient communication: A theoretical framework

### The color game

We adopt a previously proposed [4] general communication framework which takes an information-theoretic perspective via a scheme involving a speaker and a listener communicating over a noisy channel. The speaker attempts to communicate a color from the domain of colors *U*. The speaker wishes to communicate about a specific color *c* ∈ *U*, and she represents that object in her own mind as a probability distribution

$$\begin{array}{c} s=\delta (c) \end{array}$$ (1)

over the universe *U*, with mass concentrated at *c*. The speaker then utters the word *w* using a policy corresponding to a distribution *p*(*w*∣*c*) to convey this mental representation to the listener. Upon hearing this word, the listener attempts to reconstruct the speaker’s mental representation (*s*) using information conveyed in the word used by the speaker. The listener reconstruction is in turn represented by the probability distribution

$$\begin{array}{c} \ell =p(c|w),c\in U \end{array}$$ (2)

To enable us to later compare artificial languages to real languages, we will now define a number of efficiency measures that has previously been shown to be important for human languages [4, 5].

### Information-theoretic communication loss

Though the goal of the communication game is to perfectly transmit information, there are several challenges (e.g., limited vocabulary, noisy limited-bandwidth communication medium, and differences in word definitions between speakers) that make this goal impossible in reality. We take a semantic system to be informative to the extent that it yields low communication cost which can be estimated using one of the following related methods.

#### Expected surprise based on empirical estimation

The information loss can be defined as the listener’s expected surprise [5], i.e., the surprise incurred by the listener when the actual color tile that the sender encoded as a word is revealed to the listener. The expected surprise for one color tile *c* is computed as

$$\begin{array}{c} E_{c}^{ES}≔-\sum_{w\in W}p\left(w|c\right)\;{\text{log}}_{2}p\left(c|w\right). \end{array}$$ (3)

The probability distribution *p*(*w*|*c*) can be obtained in several different ways. In Gibson et al. [5], *p*(*w*|*c*) was empirically estimated from the WCS data by computing the fraction of respondents that choose to use a particular word for a given tile *c*, however, when evaluating artificial languages this is not always as easy. Fortunately, we can query the artificial agents after training, in analog to the WCS interviews, to estimate *p*(*w*|*c*). Finally, rather then separately estimating *p*(*c*|*w*), this can be computed using Bayes theorem as

$$\begin{array}{c} p\left(c|w\right)=\frac{p(w|c)p(c)}{\sum_{c^{\prime }\in U}p\left(w|c^{\prime }\right)p\left(c^{\prime }\right)} \end{array}$$ (4)

where *p*(*c*) is taken to be uniform. In this case *p*(*c*|*w*) can be seen as a Bayesian decoder.

#### KL divergence using mode map based estimation

An alternative approach, suggests the use of the KL divergence between the speaker distribution *s* and the listener distribution *l* [4], i.e.,

$$\begin{array}{c} E_{c}^{KL}=D_{KL}\left(s\left(c\right)||l\left(w\right)\right), \end{array}$$ (5)

as the measure of information loss. In the case of discrete distributions, where *s* has all its probability mass concentrated on one meaning, and *l*(*w*) = *p*(*c*|*w*) this becomes

$$\begin{array}{c} E_{c}^{KL}=-{\text{log}}_{2}p\left(c|w\right). \end{array}$$ (6)

Though *p*(*c*|*w*) can be estimated empirically for, e.g., the WCS data, it may also be computed directly from a color space partitioning [4]. This method gives us a measure of the communication cost of using a given semantic system to communicate about this domain, i.e., the distributions are derived from a mode map over *U*. More specifically, *p*(*c*|*w*) is computed as

$$\begin{array}{c} p\left(c|w\right)=\frac{\sum_{j\in Cat(c)}sim\left(c,j\right)}{\sum_{i\in U}\sum_{j\in Cat(i)}sim\left(i,j\right)} \end{array}$$ (7)

which is motivated by an exemplar selection argument (i.e., from a category); one tends to select the most representative exemplar. *Cat*(*c*) refers to the category/partition that *c* belongs to, and *sim*(*i*, *j*) measures the similarity between two colors *i* and *j* which is standard in these studies as in Regier et al. [24]:

$$\begin{array}{c} sim\left(i,j\right)≔\text{exp}(-c*dist{\left(i,j\right)}^{2}), \end{array}$$ (8)

In Eq 8, the CIELAB distance is represented as *dist*(*x*, *y*) for colors *x* and *y*. In all the simulations we report, we set *c*, the scaling factor, to 0.001 as in Regier et al. [27]. As pointed out by a reviewer, the similarity function (sim) may be interpreted as a Gaussian likelihood in CIELAB space with variance defined by s. When *x* = *y* (identical chips), the maximum value 1 is attained. As the distance between the chips grows, the value of the function falls rapidly to 0. What does this mean in qualitative terms? It means that there is a point at which the colors look so different that no noticeable additional dissimilarity effect can be distinguished.

It is interesting to note that if *p*(*w*|*c*) is taken to be a distribution with all its probability mass concentrated on the word that corresponds to the partition that *c* belongs to (which is natural given how the distribution *s* is constructed), then $E_{c}^{KL}$ can be derived from $E_{c}^{ES}$ as $E_{c}^{ES}-\sum_{w\in W}p\left(w|c\right)\;{\text{log}}_{2}p\left(c|w\right)=-{\text{log}}_{2}p\left(c|w\right)=E_{c}^{KL}$. Hence, the main difference between the two is how the distributions are estimated.

#### Aggregate measure of the communication cost

To get an aggregate measure of the reconstruction error over all colors in the domain universe of colors, we compute the expected communication cost it was noted by a reviewer that this measure is equivalent to the conditional entropy H[CjW] incurred when transferring color information between two agents over a linguistic communication channel as

$$\begin{array}{c} E≔\sum_{c\in U}p\left(c\right)E_{c}. \end{array}$$ (9)

Where *E*_*c*_ corresponds to either $E_{c}^{KL}$ or $E_{c}^{ES}$ and the need probability *p*(*c*) may be taken to be uniform [4, 5] or more informed [22]. However, all experiments in this paper use a uniform need probability.

### Well–formedness

A different criterion for evaluating the quality of a partition of the color space is the so-called *well–formedness* criterion [27]. In fact this criterion is exactly the same as the maximizing agreements objective of the *correlation clustering* problem discussed extensively in the theoretical computer science literature [31, 32]. Given the CIELAB similarity measure, we consider a graph *G* on the tiles and assign the weight $sim\left(x,y\right)-\frac{1}{2}$ on the edge connecting tiles *x* and *y*. Thus similar tiles (with similarity exceeding 1/2) will have a positive weight while dissimilar tiles (with similarity less than 1/2) will carry negative weights on the corresponding edges. The objective is then to find a clustering to maximize the weights on edges within clusters. For a given partition, we can compute this sum over all intra-cluster edges and compare it to the optimum over all partitions. While this optimum may be approximated using an heuristic approach [27], we have used an algorithm with guaranteed convergence to optima.

### Reinforcement learning framework for communication over a noisy channel

We develop a version of the general communication setup, i.e. The color game, as two automated agents trained via reinforcement learning. Our framework offers two different training approaches.

In the first training approach the agents are allowed to use continuous real valued messages during training in order to enable faster training. After training the agents are however evaulated using discrete messages. In the second approach the agents are both trained and evaluated using discrete messages.

**Fig 3:**
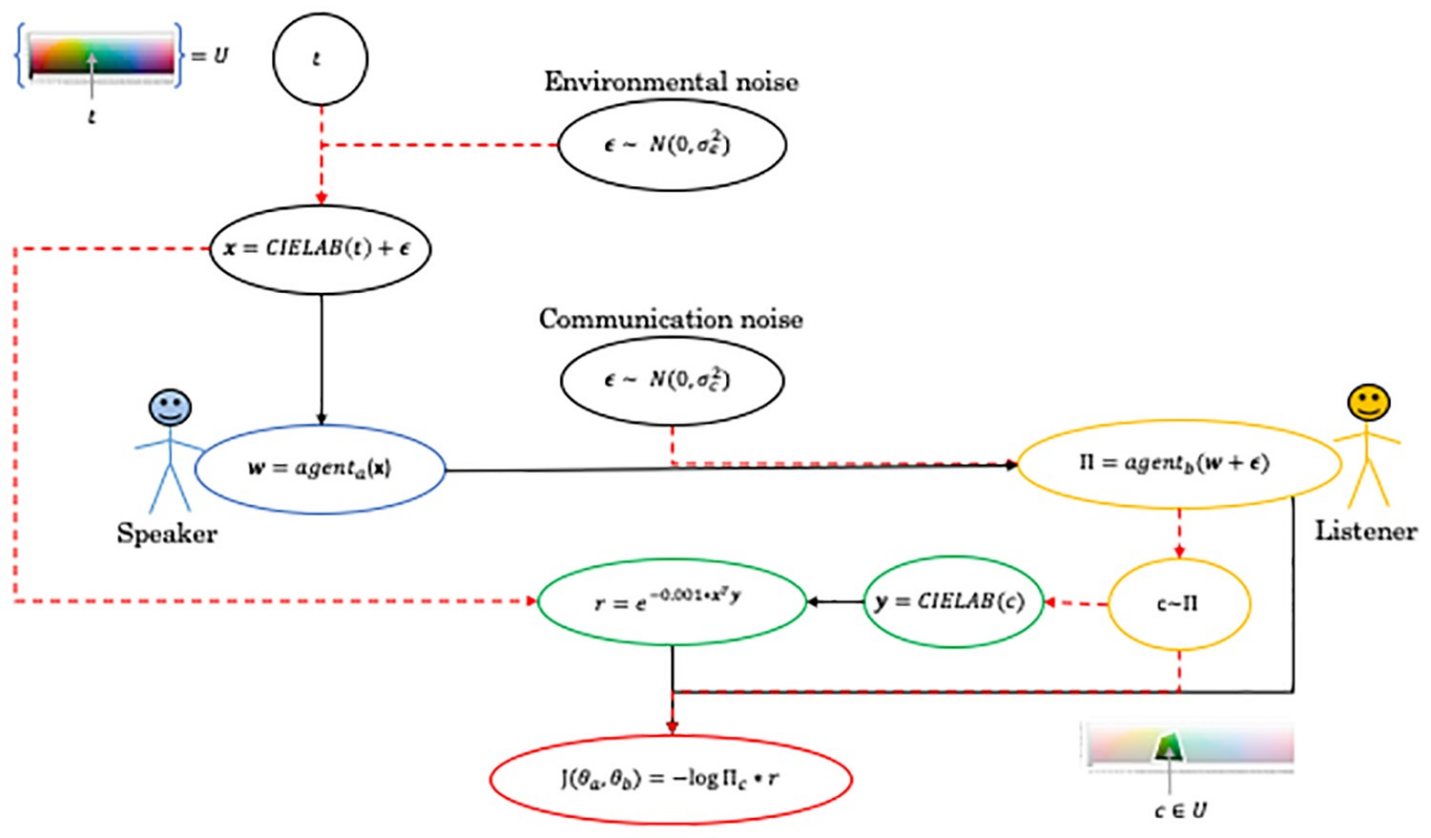
An overview showing each computation step in the model, while using continuous real valued messages during training, for one instance of the color game. Black solid arrows indicate a differentiable relation while red dashed arrows indicates a non-differentiable relation. The color of the ovals are used to highlight the different parts of the model where black is the model input, blue and yellow the agents, green the reward system, and red the reinforce cost function.

**Fig 4:**
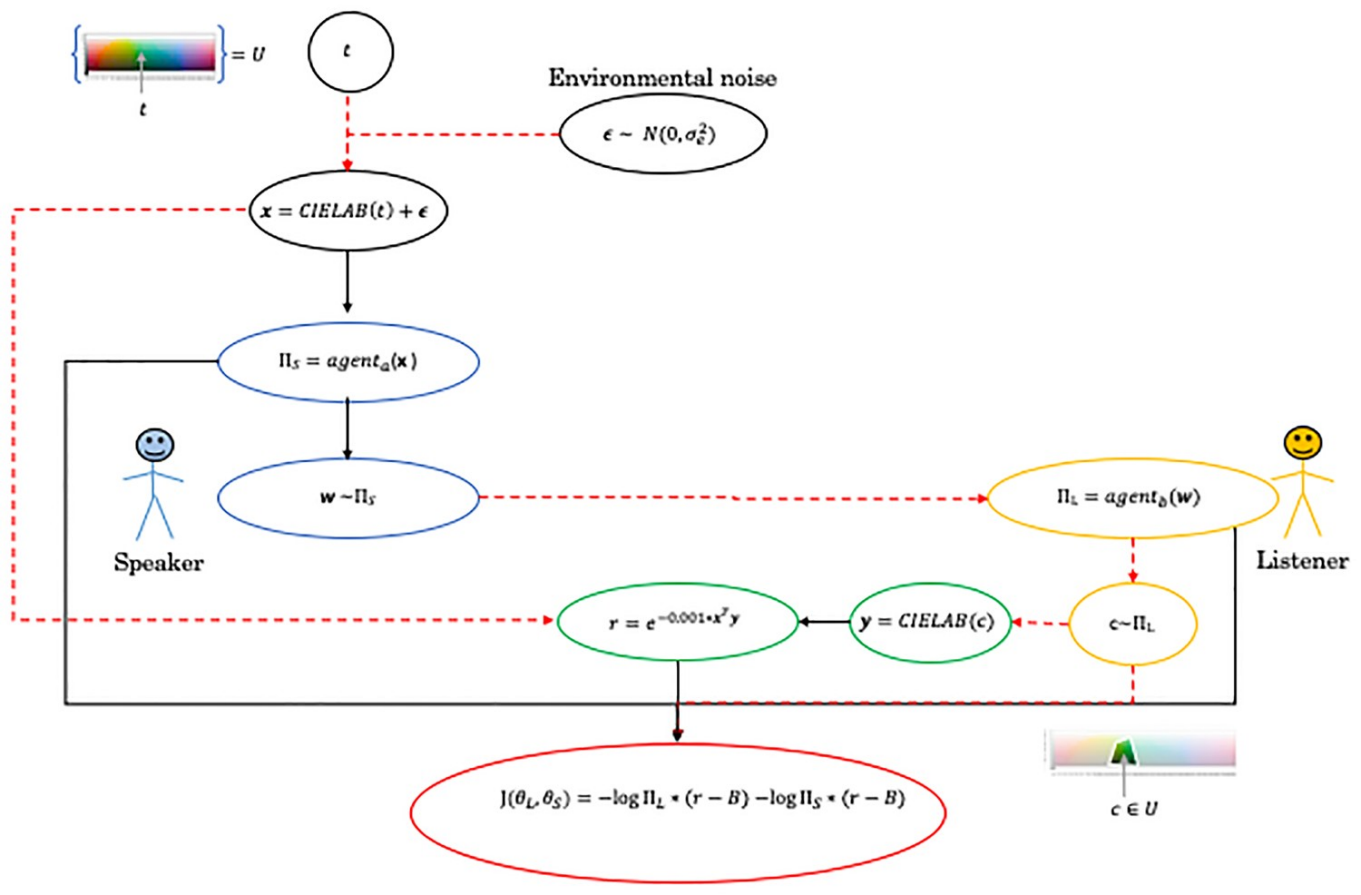
An overview showing each computation step in the model, while using discrete messages during training, for one instance of the color game. Black solid arrows indicate a differentiable relation while red dashed arrows indicates a non-differentiable relation. The color of the ovals are used to highlight the different parts of the model where black is the model input, blue and yellow the agents, green the reward system, and red the reinforce cost function.

An overview of the model trained with continuous real valued messages is shown in Fig 3, the model trained with discrete messages is shown in Fig 4. Note that the main difference between the training approaches is whether the communication channel is differentiable, black solid arrows, or not, red dashed arrows.

It turns out that training with discrete messages is more time consuming and it becomes harder for the agents to converge and agree on a certain color partition. Most our analysis will therefore be with respect to agents trained with continuous real valued messages and it can be assumed that continuous real valued messages was used during training if nothing else is stated. However, we also provide a section where we compare a limited number of experiments ran with discrete messages to their corresponding continuous real valued counterpart.

#### Continuous policy

The sender trying to communicate the target color *t* ∈ *U* creates a word vector

$$\begin{array}{c} w=\text{softmax}\left(\phi_{s}^{T}\text{ReLU}\left(\theta_{s}^{T}\left[\text{CIELAB}\left(t\right)+\epsilon_{e}\right]\right)\right),\epsilon_{e}∼N\left(0,\sigma_{e}^{2}\right). \end{array}$$ (10)

where ${\text{softmax}}_{j}\left(z\right)=e^{z_{j}}/\sum_{i}^{\left|z\right|}e^{z_{i}}$, ReLU(*z*) = max(0, *z*), {***ϕ***_*s*_, ***θ***_*s*_} are the parameters of the sender agent, and ***ϵ***_**e**_ model environment noise. **w** is subsequently sent to the listener agent over a noisy communication channel as

$$\begin{array}{c} m=w+\epsilon_{c},\epsilon_{c}∼N\left(0,\sigma_{c}^{2}\right). \end{array}$$ (11)

Please note that, though this message will start out as a continuous real valued message the noise will make it converge, as training goes on, to a peaked distribution with almost all probability mass concentrated to one dimension for each color [17]. Further, when we extract the final resulting language we use discrete *m* vectors as, i.e. where all dimensions but one is zero, to ensure that no extra information is encoded.

The receiver interprets the message received (**m**) and computes a distribution over all colors in *U* as

$$\begin{array}{c} p\left(U|m\right)=\text{softmax}(\phi_{r}^{T}\text{ReLU}\left(\theta_{r}^{T}m\right)). \end{array}$$ (12)

By now merging Eqs (10), (11), and (12) we get the final policy

$$\begin{array}{c} \Pi_{\Omega }\left(U|t\right)=p\left(U|t\right) \end{array}$$ (13)

where $\Omega =\{\theta_{s}\in R^{k\times 3},\phi_{s}\in R^{d\times k},\theta_{r}\in R^{k\times d},\phi_{r}\in R^{|U|\times k}\}$ parameterizes the entire model. The sender and receiver agents are modeled using *multilayer perceptrons*, bias terms have been omitted for brevity, with one hidden layer of *k* = 20 units, and the size of the message vector is set to *d* = 50 for all experiments. Note that *d* will set the maximum number of color terms that the system can use to 50; however, this is far above what is used in practice and not what will determine the number of terms actually used by the system.

#### Cost function for continuous policy

Finally, plugging the policy and reward into REINFORCE [33] we get the cost function

$$\begin{array}{c} J\left(\Omega \right)=-\frac{1}{N_{b}}\sum_{n}^{N_{b}}\text{log}\Pi_{\Omega }\left(U=c_{n}|t\right)*r_{n}. \end{array}$$ (14)

where *N*_*b*_ corresponds to the number of games over which the cost is computed and *r*_*n*_ is the reward detailed in section Reward. For more on REINFORCE please see the section describing Materials and methods.

### Discrete policies

In order to use discrete communication during training a message **m**, represented as a discrete vector where all but one dimension is equal to zero, is sampled from the categorical distribution over the set of possible color terms

$$\begin{array}{c} \text{m}\sim p\left(W|t\right)=\text{softmax}\left(\phi_{s}^{T}\text{ReLU}\left(\theta_{s}^{T}\left[\text{CIELAB}\left(t\right)+\epsilon_{e}\right]\right)\right)\\ \epsilon_{e}\sim N\left(0,\sigma_{e}^{2}\right) \end{array}$$ (15)

Eq (15) gives us the policy of the sender

$$\begin{array}{c} \Pi_{\Omega_{s}}\left(W|t\right)=p\left(W|t\right) \end{array}$$ (16)

where $\Omega_{s}=\left\{\theta_{s}\in R^{k\times 3},\phi_{s}\in R^{d\times k}\right\}$, under discrete communication.

Further, the receiver interprets the received message (**m**) and computes a distribution over all colors *U* as described in Eq (12). Hence, the receiver policy becomes

$$\begin{array}{c} \Pi_{\Omega_{r}}\left(U|m\right)=p\left(U|m\right) \end{array}$$ (17)

where $\Omega_{r}=\left\{\theta_{r}\in R^{k\times d},\phi_{r}\in R^{|U|\times k}\right\}$.

As in the case with the continuous policy, the sender and reciever will be modelled using *multilayer perceptions* with one hidden layer consisting of *k* = 20 units. The size of the message vector is set to *d* = 50.

#### Cost functions for discrete policies

Furthermore, due to the non-existence of a gradient over the communication channel we end up with two distinct policies, one for the sender and one for the receiver, which require us to have two cost functions that will be optimized simultaneously.

As a result, the cost function for the sender becomes

$$\begin{array}{c} J\left(\Omega_{s}\right)=-\frac{1}{N_{b}}\sum_{n}^{N_{b}}\text{log}\Pi_{\Omega_{s}}\left(m_{n}|t\right)*\left(r_{n}-B_{n}\right) \end{array}$$ (18)

and one for the receiver it becomes

$$\begin{array}{c} J\left(\Omega_{r}\right)=-\frac{1}{N_{b}}\sum_{n}^{N_{b}}\text{log}\Pi_{\Omega_{r}}\left(U=c_{n}|t\right)*\left(r_{n}-B_{n}\right). \end{array}$$ (19)

Here the term *B*_*n*_ is the running mean of the rewards acquired so far and is used as a baseline. Introducing a baseline to the cost function is a standard procedure used to reduce the inherent high variance in the REINFORCE algorithm [34] and we add this baseline to cope with the difficulties induced by using discrete messages.

Since there is no gradient over the communication channel the policy update of one agent will be independent of the policy update of the other agent. Thus, the environment will be non-stationary and it will be harder for the agents to agree on a certain color partition and converge [35].

### Reward

When training the model the computed policy is used to sample a guess

$$\begin{array}{c} c∼\Pi_{\Omega }\left(U|t\right) \end{array}$$ (20)

which is in turn used to compute a reward *r* that reflects the quality of the guess in respect to the target color *t*.

$$\begin{array}{c} r≔\text{sim}(c,t) \end{array},$$ (21)

where sim is the color similarity function defined in Eq 8.

#### Comment

One could think of the reward in the setting of the sender and the receiver attempting to solve a task co-operatively. Suppose that in the process, they need to communicate the color. Then, presumably, their success in carrying out the task is related to how well the color decoded by the receiver approximates the color the sender intended to transmit. Thus, it is reasonable to assume that the reward corresponding to how well they succeed in carrying out the task is proportional to the similarity of the decoded color to the one the sender intended to convey. One could argue the reward above is a good proxy for the reward corresponding to successfully carrying out the task co-operatively.

### Training

All parameters are initialized to random values and trained using stochastic gradient decent with ADAM [36]. The batch size when training with continuous real valued messages is set to *N*_*b*_ = 100 games, and the model is trained for a total of *N* = 20000 episodes.

Moreover, when using discrete communication in the training step we set the batch size to *N*_*b*_ = 256 and the two models are trained for *N* = 25000 episodes. We have to increase the number of episodes and the batch size, compared to the case with a continuous real valued, in order to handle the increased difficulty induced by the discrete communication. All other parameters are set to the same value used for training with continuous real valued messages.

### Generate partitioning

After training the agents a color-map, characterising the emerged communication schema, is constructed. This is accomplished, in analog to the WCS, by asking the speaking agent to name (or categorize) each color-tile as

$$\begin{array}{c} cat\left(t\right)=\underset{i}{\text{argmax}}\;w_{i}\left(t\right), \end{array}$$ (22)

where *w*_*i*_(*t*) is the ith element of the message vector **w**, defined in Eq (10), as a function of the color-tile (*t*) shown to the agent.

## Efficiency analysis

Based on recent results [4, 5] showing that communication tends to be efficient, we would like to investigate whether the communication schema that emerges between reinforcement learning agents exhibits similar traits. In order to evaluate this, we compare the reinforcement learning agent languages to the languages of the WCS in terms of the communication cost, defined in Eq (9), and the related criterion described under Well–formedness in the Materials and methods section. This comparison is done in buckets of the number of color terms used, where a higher number of words is expected to result in lower communication cost. To provide the reader with a sense of scale, we compliment this picture with results using (1) a random partitioning with a uniform distribution of tiles per color word and (2) the correlation clustering of the tiles in CIELAB space; for more details, CIELAB correlation clustering in Materials and methods. These baselines are not to be interpreted as competing models but rather an upper and lower bound on the achievable efficiency. We have left for future work another relevant baseline to which we could have compared our systems and which may set a higher bar for the comparison, as suggested by a reviewer: the rotational baseline [27], i.e., a communication schema derived by rotating the partitioning of a real language.

### Discrete vs continuous RL training

In order to justify the use of continuous real valued messages during training, we perform a comparison between training with continuous real valued and discrete messages by computing the adjusted Rand index for the resulting partitions; see Table 1. (See the Materials and methods section for a short explanation of adjusted Rand index).

**Table 1:** Comparison between continuous real valued messages during training and discrete messages during training.

Terms	H-H	DM-DM	RVM-RVM	DM-RVM
3	.701(±.051)	.334(±.026)	.273(±.034)	.303(±.026)
4	.452(±.031)	.397(±.023)	.337(±.028)	.323(±.024)
5	.476(±.018)	.459(±.018)	.373(±.023)	.376(±.015)
6	.528(±.011)	.524(±.006)	.537(±.033)	.485(±.009)
7	.472(±.016)	.549(±.003)	.593(±.028)	.544(±.006)
8	.471(±.041)	.505(±.007)	.518(±.017)	.484(±.007)
9	.584(±.057)	.457(±.023)	.510(±.007)	.472(±.009)
10	.718	.443	.549(±.008)	.505(±.015)

Abbreviations used in table column headers: H = human, RVM = reinforcement learning training with continuous real valued messages and DM = reinforcement learning training with discrete messages. Value within parenthesis indicate a 95% confidence interval. The row corresponding to 11 color terms was excluded since no such partition was generated when training with discrete messages.

We observe a high adjusted Rand index between training with the two different message types (DM-RVM), which indicates that the two training approaches result in partitions with a fair amount of similarity. In addition, their corresponding internal consistency, (DM-DM) and (RVM-RVM), seems to be on the same level as the internal consistency of human partitions (H-H). The only major difference seems to be for 3 and 10 color terms, but as previously stated, these color terms are outliers when it comes to human partitions. The main difference between the two different training models is that the discrete model takes much longer to train. Hence, in most of the rest of the paper, we report results based on the continuous training model; as indicated above, the results are quite robust to the two different modes of training. In section Quantitative similarity using adjusted Rand index, we again give an explicit comparison of results using the two different methods of training.

**Fig 5:**
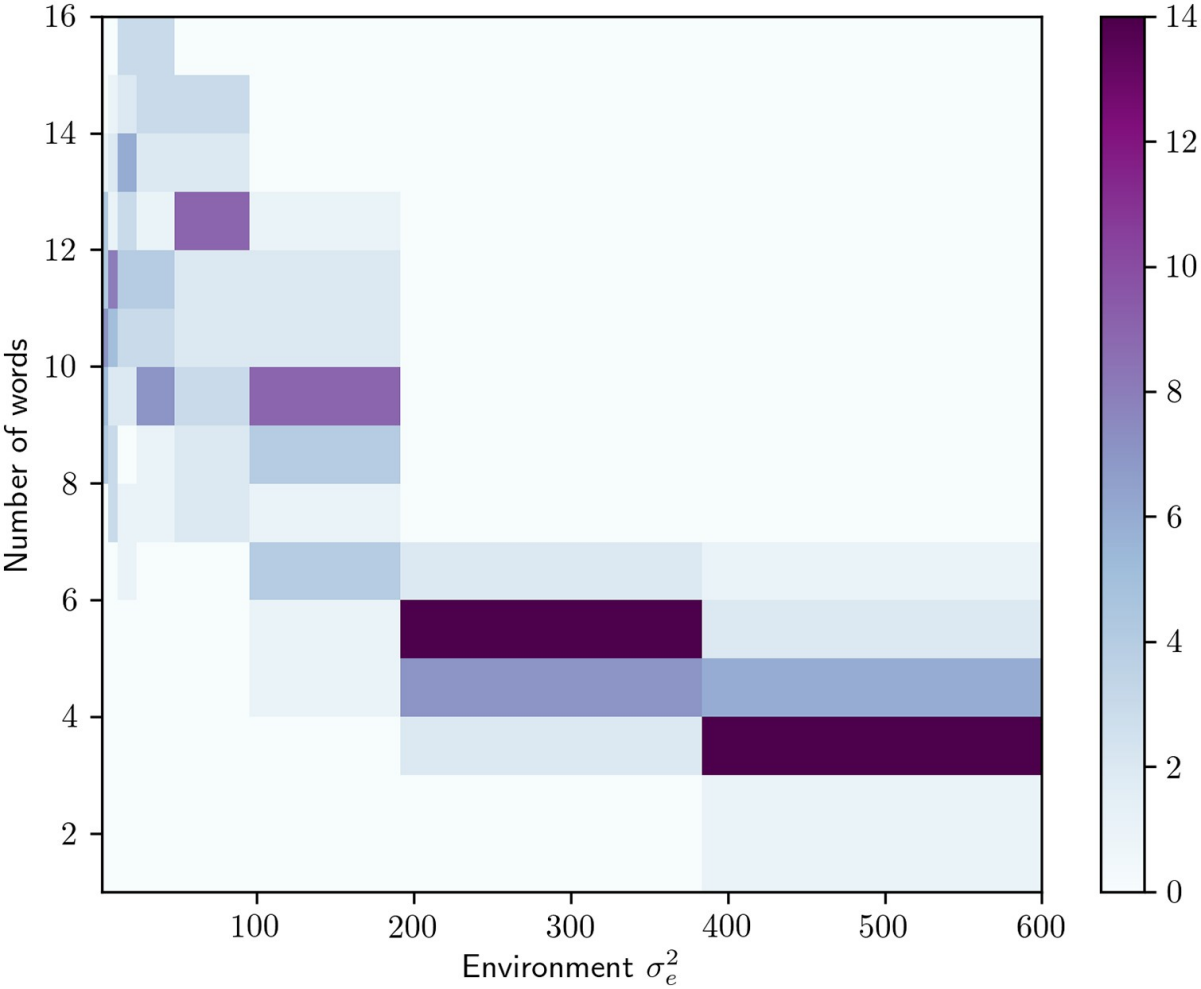
2D histogram showing the number of emerged communication systems that end up using a particular number of color terms when trained using a particular amount of environmental noise. 25 experiments are run for each level of noise $\sigma_{e}^{2}\in \left\{1,2,4,8,16,32,64,128,256,512\right\}$. Hence, each bin on the x axis shows the distribution over number of words resulting from that level of noise.

The RL agents are trained while applying a varying amount of environmental noise $\sigma_{e}^{2}\in \left\{1,2,4,8,16,32,64,128,256,512\right\}$, i.e. Gaussian noise added to the color chips in CIELAB space, and the results are averaged over 250 experiments (25 for each level of noise). The variation in environmental noise encourages the model to find solutions with varying numbers of color terms used, see Fig 5, an approach that stands in stark contrast to modeling the language giving a static number of color terms, e.g. [4], and allows us to investigate what environmental properties affect the size of the color vocabulary. The level of communication noise was kept constant at $\sigma_{c}^{2}=0.1$ for all experiments.

### KL loss evaluation

**Fig 6:**
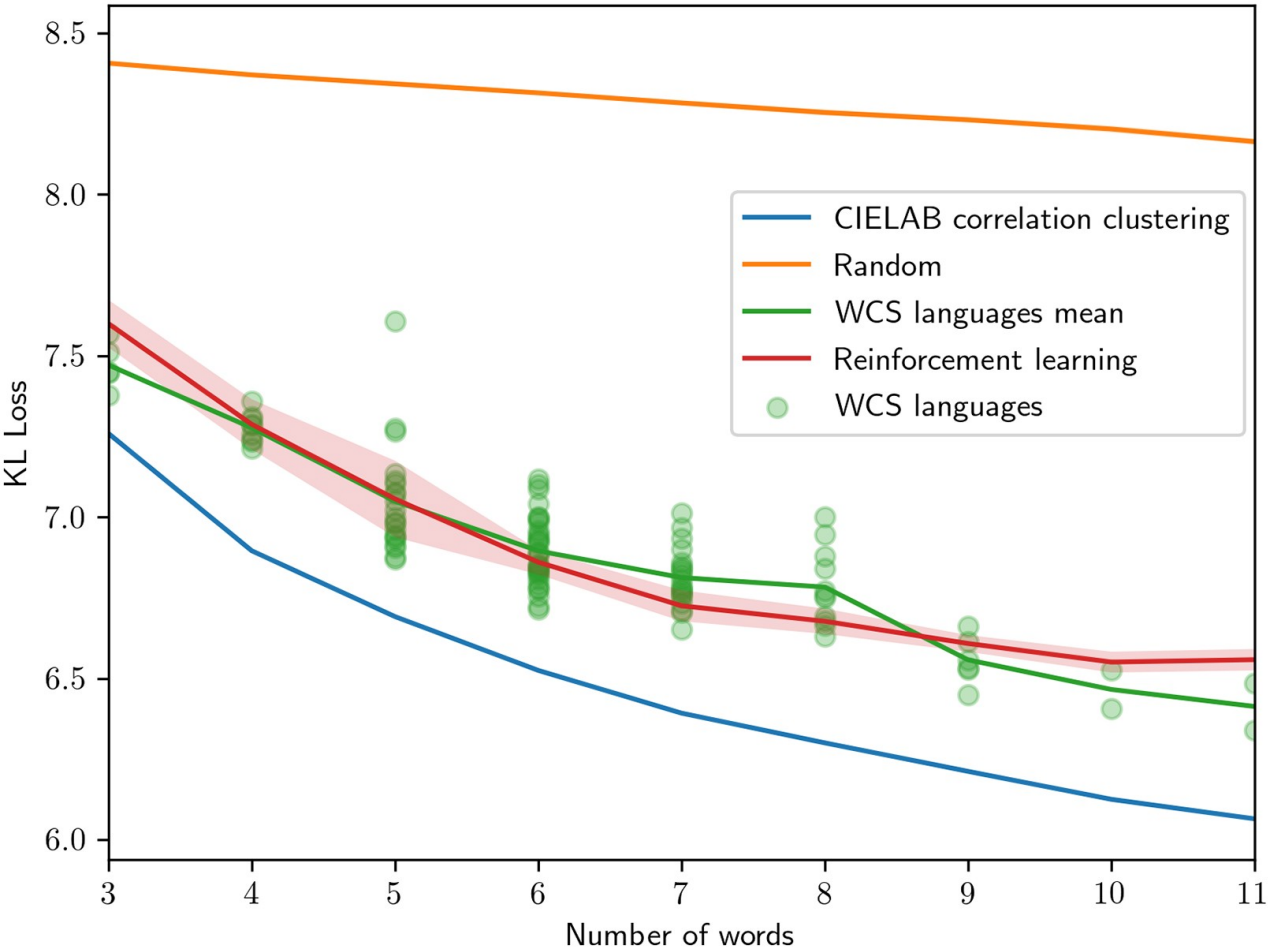
KL loss for varying number of color words used. The circles indicate the KL loss of individual WCS languages sorted based on the number of color words used. The shaded regions indicate a 95% confidence interval. Note, the WCS language data points is a reproduction from [4].

The results in terms of KL loss, defined in Eq (6), can be seen in Fig 6. The WCS language data are shown both as individual languages, shown as rings, and the mean of all languages. The other results are presented as means with a 95% confidence interval indicated as a colored region. As previously shown, human languages are significantly more efficient than chance but do not reach perfect efficiency [4], here approximated by CIELAB CC. Further, the partitions produced by the reinforcement learning agents closely follow the efficiency of the human languages of the WCS.

### Expected surprise evaluation

**Fig 7:**
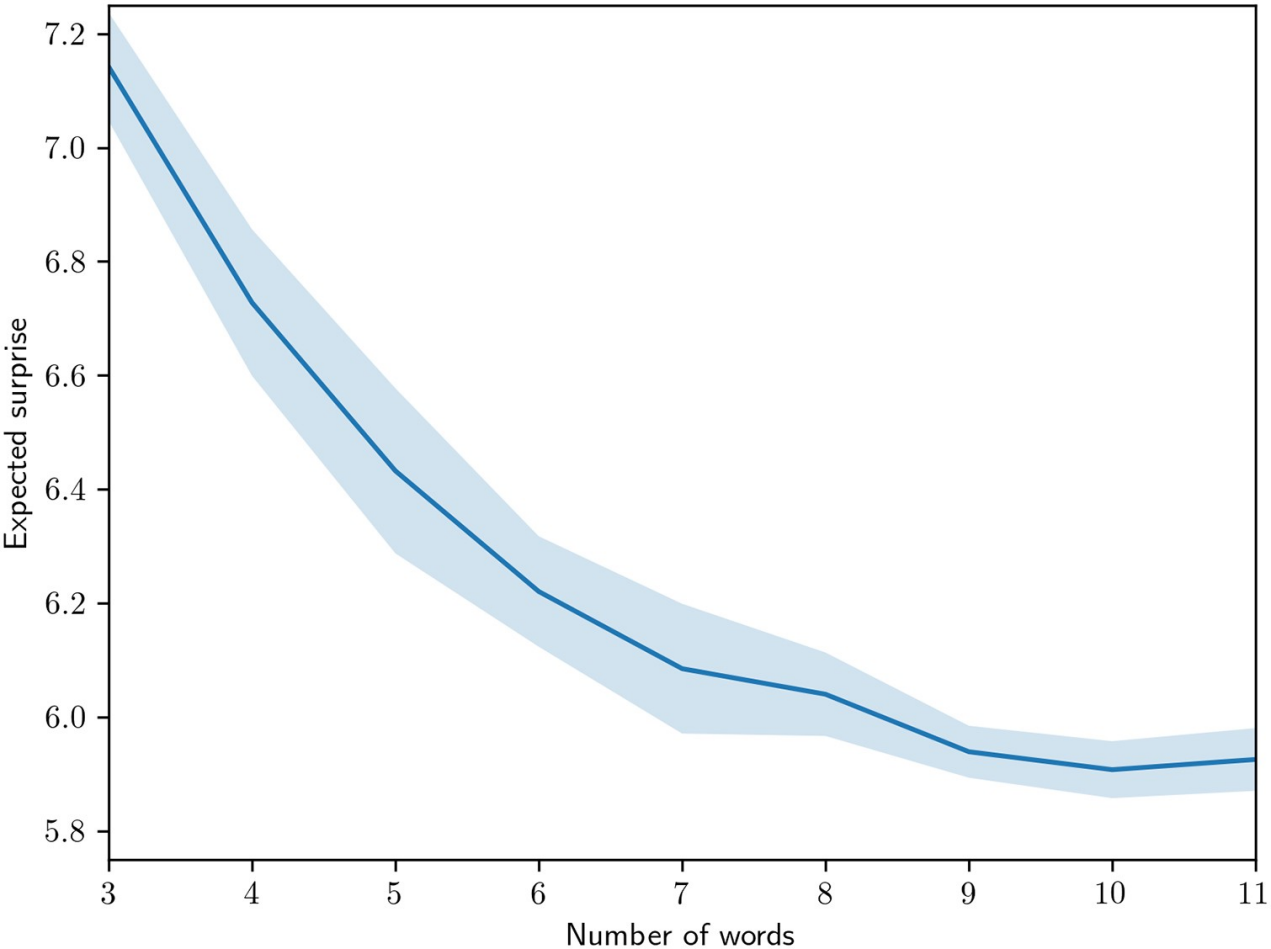
Expected surprise for varying number of color terms used. The shaded regions indicate a 95% confidence interval.

Fig 7 show the expected surprise, defined in Eq (3), resulting from the same experiment. These results are consistent with previously reported results in experiments with human subjects [5].

### Well–formedness evaluation

**Fig 8:**
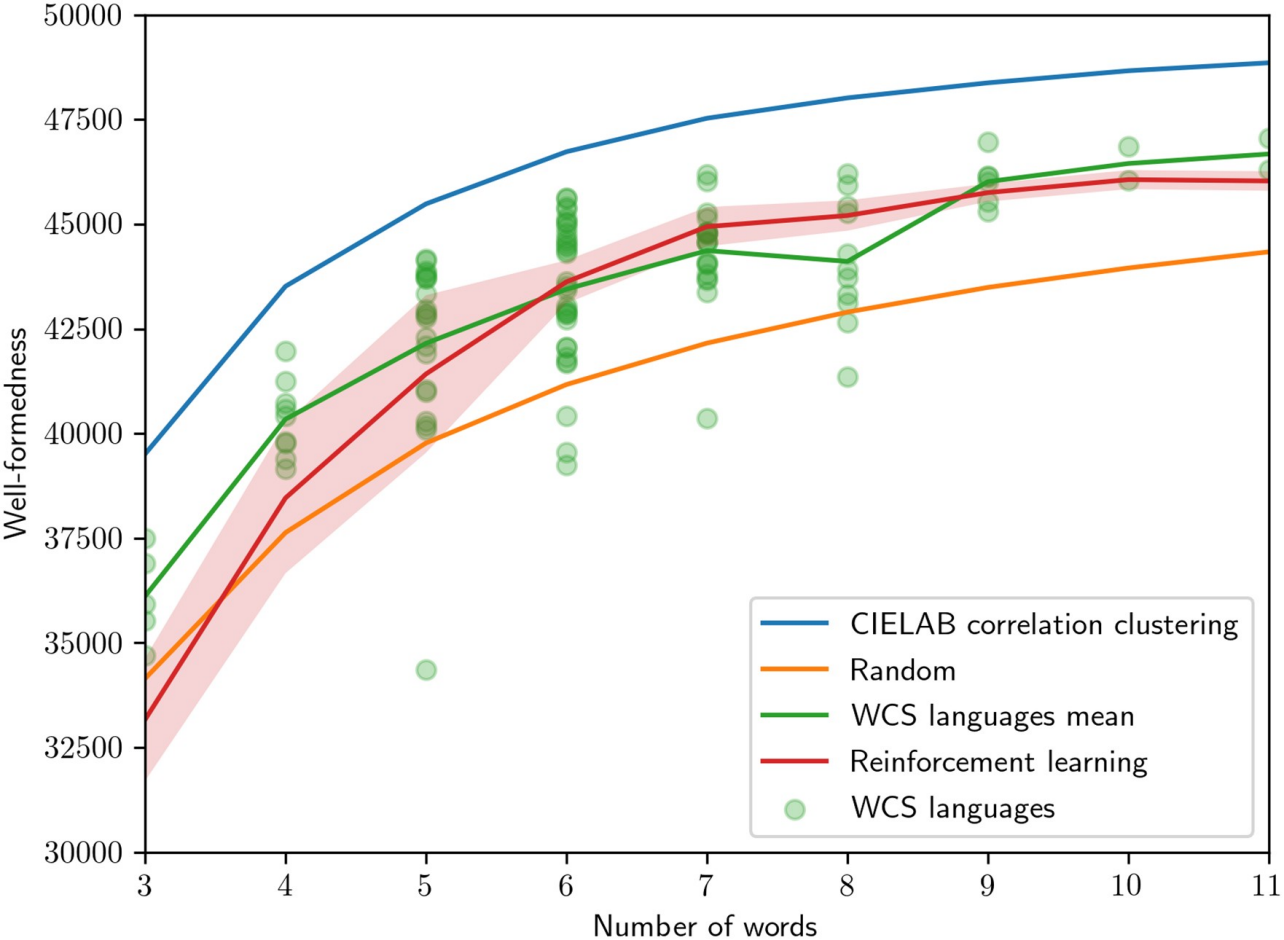
Well–formedness for varying number of color words used. The circles indicate the well–formedness of individual WCS languages sorted based on the number of words. The shaded region indicates a 95% confidence interval.

In Fig 8 we show the value of the well–formedness objective, for each number of color terms. The top line represents the optimal value corresponding to the optimal partition computed by correlation clustering. The remaining lines show the value attained by partitions produced by our reinforcement learning algorithm and by WCS languages. We observe that the RL partition is close to the optimal partition, and several human languages are clustered around this. Most of these are significantly better than the value for a random partition. These results are consistent with results from experiments with human subjects [27].

## Partitioning characteristics

In order to further evaluate the human resemblance of our artificially-produced color space partitions, we compare a range of color maps both qualitatively and quantitatively. The quantitative comparison is done using adjusted Rand index.

### Quantitative similarity using adjusted Rand index

In order to get a sense of scale, we start by computing the internal Rand index for the reinforcement learning agents and the WCS languages; see Table 2. This is accomplished by averaging the Rand index between all objects within the group. Comparing the internal consistency of human and RL partitionings, it seems to be on a similar level for most numbers of terms but differs for the 3 color term and 10 color term levels where the human languages yield a higher index. However, it should be noted that there are very few samples behind the human figures for those groups (i.e., 4 languages with 3 color terms and 2 with 10), and that they are outliers compared to the others. Subsequently, we compute the average Rand index across different groups, and by comparing these numbers, we can get a sense of their level of similarity; see Table 2. We observe fair amounts of similarity, and the human partitions are more similar to the CIELAB partitions than to the RL partitions, but the RL partitions are more similar to the CIELAB partitions.

**Table 2:** Comparison of the human languages in WCS to generated languages using Rand index.

Terms	H-H	RL-RL	H-RL	H-CCC	RL-CCC	H-R
3	.701(±.051)	.273(±.034)	.173(±.028)	.385(±.038)	.192(±.020)	0(±.000)
4	.452(±.031)	.337(±.028)	.167(±.019)	.273(±.020)	.319(±.023)	0(±.000)
5	.476(±.018)	.373(±.023)	.223(±.015)	.356(±.018)	.359(±.026)	0(±.000)
6	.528(±.011)	.537(±.033)	.277(±.009)	.396(±.013)	.433(±.029)	0(±.000)
7	.472(±.016)	.593(±.028)	.292(±.008)	.409(±.016)	.456(±.007)	0(±.000)
8	.471(±.041)	.518(±.017)	.281(±.010)	.330(±.018)	.419(±.011)	0(±.000)
9	.584(±.057)	.510(±.007)	.321(±.006)	.399(±.021)	.426(±.008)	0(±.000)
10	.718	.549(±.008)	.316(±.012)	.416(±.050)	.412(±.009)	0(±.001)
11	.472	.543(±.009)	.309(±.010)	.371(±.022)	.402(±.005)	0(±.001)

Abbreviations used in table column headers: H = human, RL = reinforcement learning, CCC = CIELAB correlation clustering and R = random. Value within parenthesis indicate a 95% confidence interval.

Again, the indices for the lower number of color terms are conspicuous, but this time it has to do with the RL agents that exhibit a much lower similarity for 3 and 4 terms. A possible reason for this is connected to the way we modulate the number of color words in the RL model, i.e., by adding noise to the color chips, which may have drowned out much of the CIELAB information for the very low number of color terms, which requires a large amount of noise to appear. This would explain why RL is less similar to CCC for low terms as well. This observation suggests that other mechanisms, apart from environmental noise, might influence the number of words used in human languages.

Furthermore, in Table 3, we compare the resulting partitions from the two different training approaches with color partitions from human language, (H-DM) and (H-RVM). We observe that both approaches seem to produce solutions which have the same level of similarity towards human partitions, and their corresponding 95% confidence intervals overlap for all but 5, 6 and 7 color terms. However, for this number of color terms, the corresponding adjusted Rand indices for the different training approaches are still close to each other.

**Table 3:** Comparison between continuous real valued messages during training and discrete messages during training.

Terms	H-DM	H-RVM
3	.168(±.019)	.173(±.028)
4	.184(±.011)	.167(±.019)
5	.265(±.010)	.223(±.015)
6	.301(±.004)	.277(±.009)
7	.312(±.003)	.292(±.008)
8	.286(±.006)	.281(±.010)
9	.327(±.014)	.321(±.006)
10	.286(±.111)	.316(±.012)

Abbreviations used in table column headers: H = human, RVM = reinforcement learning training with continuous real valued messages and DM = reinforcement learning training with discrete messages. Value within parenthesis indicate a 95% confidence interval. The row corresponding to 11 color terms was excluded since no such partition was generated when training with discrete messages.

In our setting, we have observed a fair amount of similarity between the resulting color partitions when training with discrete and continuous real valued messages. The resulting partitions also shows same level of similarity towards human partitions. Since it is easier and faster to train with continuous real valued messages, downstream analysis will be performed using only the training approach with continuous real valued messages.

### Analysis of consensus color partitions

#### Color partitioning across multiple human languages

**Fig 9:**
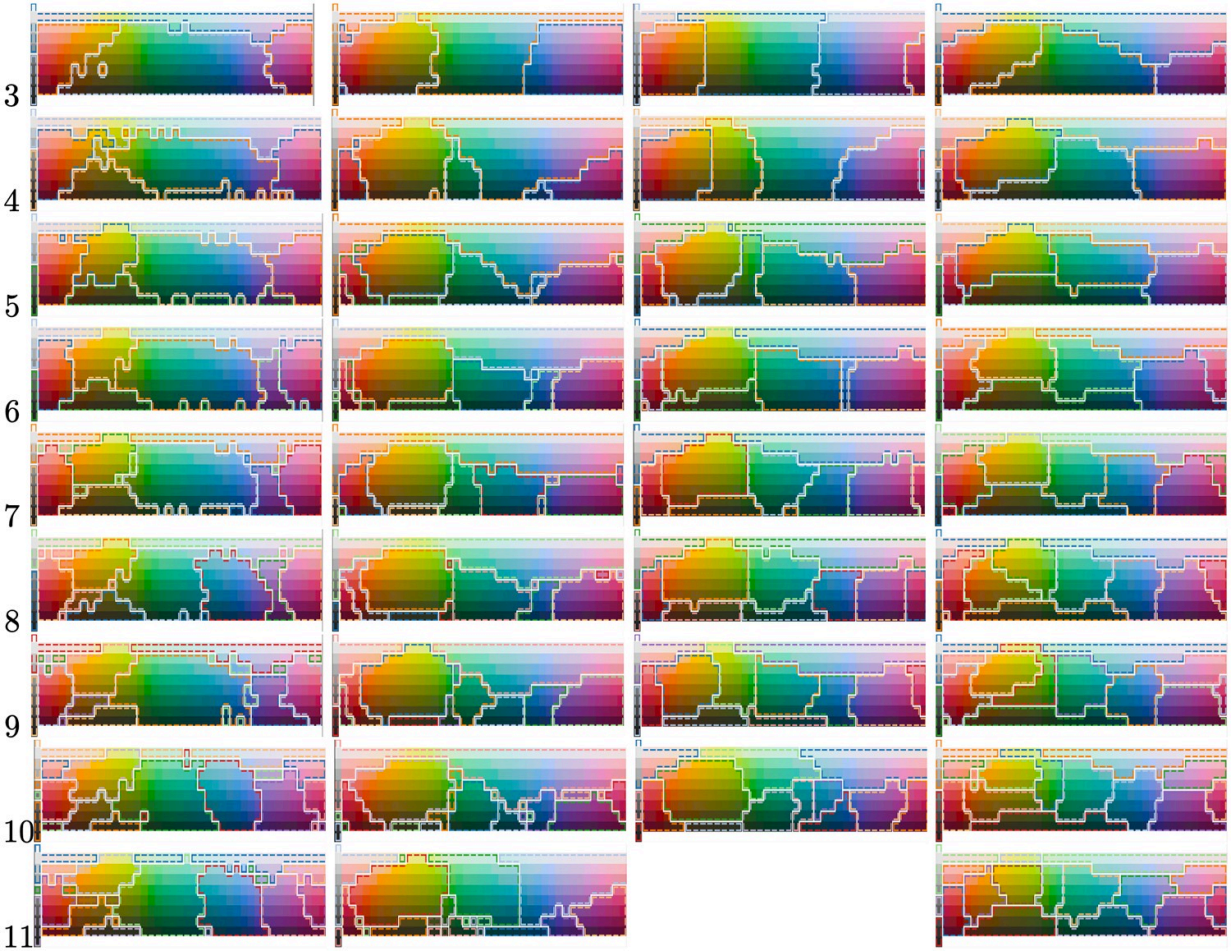
Color space partitions for different number of color terms from four different sources. The first column show human language consensus maps, i.e., consensus cross all languages in the WCS that uses the particular number of color term indicated to the left. The second column corresponds to the consensus maps over reinforcement learning partitionings using continuous real valued messages during training. The third column corresponds to the consensus maps over reinforcement learning partitionings using discrete messages during training. Finally, the fourth column of partitions is constructed using correlation clustering directly on the graph defined by the CIELAB distance between each color tile. Notice that, for 11 color terms, no partition was generated using discrete messages.

To enable qualitative comparison of human and artificial color maps, we produce one consensus color map for each number of color words where each color map is based on all the human languages in WCS with the given number of color words. The consensus map is computed using correlation clustering, described under Consensus maps by correlation clustering in Materials and methods. This process results in the 9 color maps shown to the left in Fig 9. Each of them represents a consensus color partitioning of all languages using the respective number of color words; e.g., all languages using three color terms form one consensus map.

#### Reinforcement learning consensus partitions

The same procedure, as described above, is subsequently performed for the artificial languages produced in the Efficiency analysis experiment and presented in the middle column of Fig 9. The main motivation for creating consensus maps over many experiments is to make the result more robust to variations between experiments. That said, as shown in a Table 2, the consistency between reinforcement learning experiments (RL-RL) are at a level similar to human language variation (H-H). Comparing the consensus maps of the RL model to the human consensus maps, there are many similarities, especially for the languages with many color terms. One exception is however the lack of light/dark gray separation for languages with few color terms, which is not captured in the RL maps. It is however captured in the maps with higher number of color terms, which might indicate that it has to do with the type of noise that is applied to the environment during training, which is uniform in all dimensions, something that might not be true in a natural environment. In fact, analyzing the WCS color chips, the light/dark dimension has the lowest standard deviation of the 3 dimensions, i.e., 23.3 compared to 29.0 and 32.9.

#### CIELAB correlation clustering partitions

Finally, to the right in Fig 9, we show the partitions produced by applying correlation clustering to CIELAB similarities produced in the Efficiency analysis experiment.

### Developing an artificial language

As a language develops over time, concepts tend to get refined into sub-categories; e.g., when a new color term comes into use, it tends to represent a subset of a region previously represented by another color term. It was suggested in Berlin and Kay [24] that there is an evolutionary order on the partitioning of the color space. In this proposal, the existing partitions are updated in a specific order, with the initial distinction being light/dark, followed by red, green, yellow, blue, and then other colors. The update occurs on the emergence of new color words.

**Fig 10:**
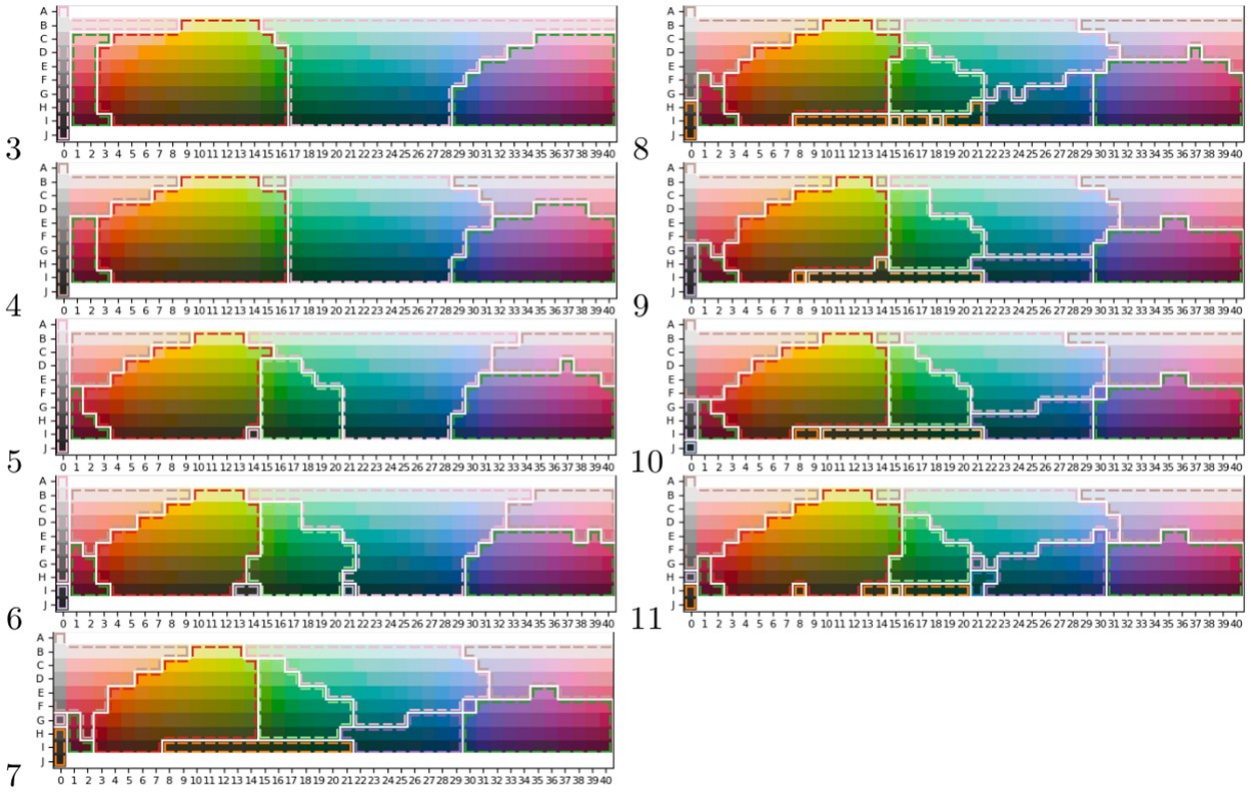
Color maps captured during one training session as the emerging language progress towards an increasing number of terms.

**Fig 11:**
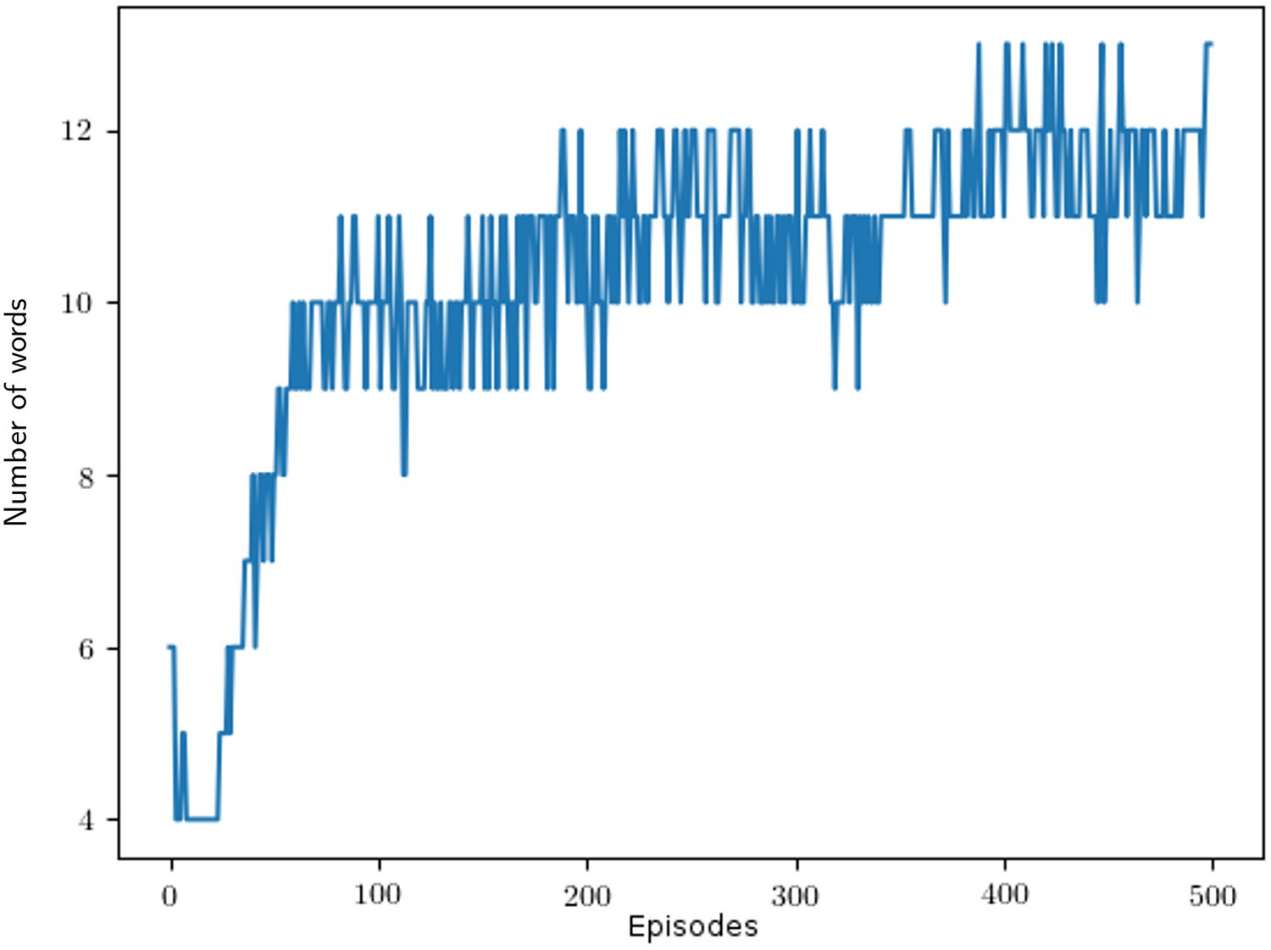
Change in the number of words used by the agents during training. The X-axis represents the number of episodes trained and the Y-axis the number of words used at that point.

**Fig 12:**
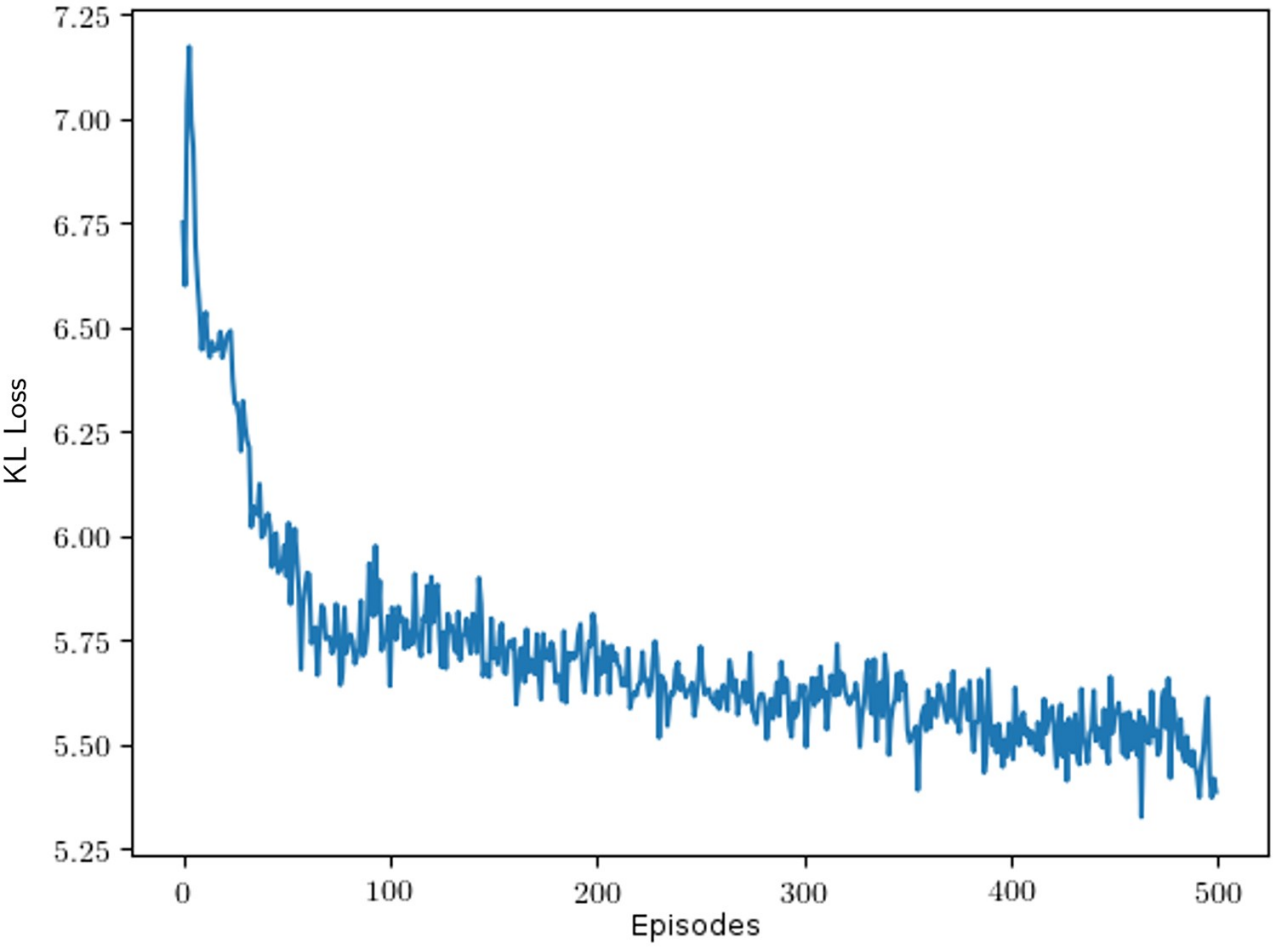
KL loss change during training. The X-axis shows the number of episodes.

To investigate whether similar patterns emerge while the languages developed between reinforcement learning agents, we show snapshots of the color partitionings as they develop during one training episode in Fig 10. To complement this picture, we show how the number of terms develops on a timeline in Fig 11 and how the KL loss falls as the number of terms used goes up on the same timeline in Fig 12. The color partition snapshots were captured on the last episode using that number of color terms. As seen in Fig 10, the order in which colors emerge in human languages is not very well replicated in the artificial language while the subdivision of partitions is captured to a greater extent. Further examining Fig 11, it is interesting to note that the number of color terms used tend to steadily go up during training—this resembles how the vocabulary of human speakers tends to grow when a community communicates frequently regarding a specific subject; e.g., people working with color tend to use a larger-than-average color vocabulary, especially when talking to each other.

## Environmental impact on partitioning

In this section we describe the results of controlling environmental factors such as the noise level in the various channels over which the agents communicate.

### Modulating the vocabulary size by varying environmental noise

**Fig 13:**
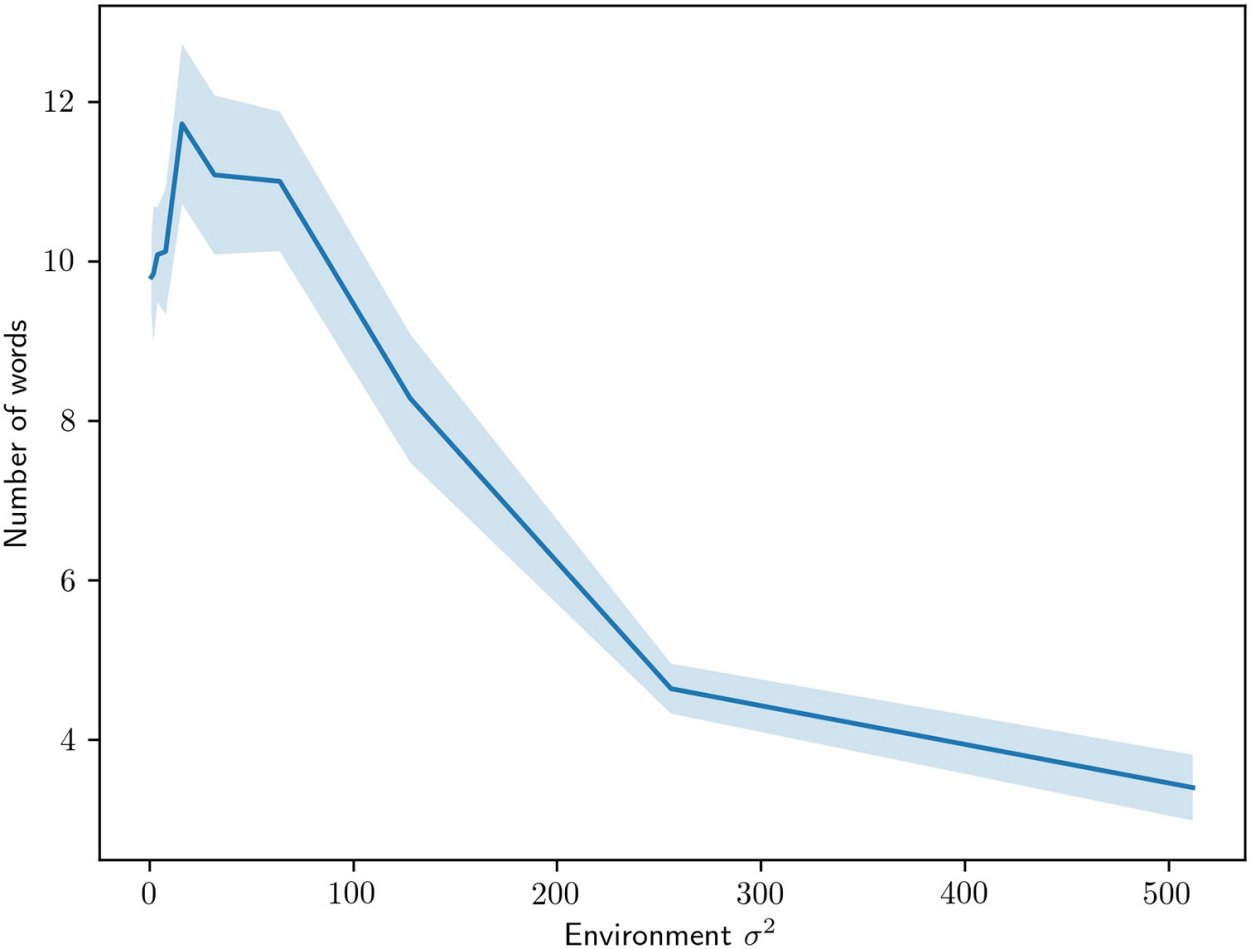
The number of color terms used by the agents when different amounts of noise are applied to their environments.

Environmental noise is noise added to the color chips before shown to the agent. In information-theoretic terms, this channel refers to the conditional probability *p*(*w*∣*c*). This emulates the fact that when referring to an object in the real world it may vary in color. This is especially true in a natural environment where, for instance, a tree may vary considerably in color over time; hence, when referring to specific trees using color, it may not be useful to develop very exact color terms. In contrast, in an industrialized society, exact color information may carry more information, which could be one reason for why they tend to use more color terms. To show the effect of varying the environmental noise on our artificially synthesized languages, two experiments are conducted:

The first experiment investigates the effect on the number of terms used as a function of environmental noise. As can be seen in Fig 13, this has the effect of lowering the number of color words of the resulting language. Though we cannot say that this effect is the main driving force behind language complexity in real languages, it is clear that it can have a significant effect in a setting like ours. An interesting effect that we have seen consistently is that low levels of environmental noise increase the size of the vocabulary in the resulting language.

The second experiment measures to what extent the noise affects how the space is partitioned, apart from the number of terms used. The experiment is conducted by computing, for each number of color terms used, the internal consistency between all partitionings that resulted in that number of terms regardless of the level of noise and the average internal consistency between partitionings created using the same level of noise. The environmental noise levels used in this experiment are ***ϵ***_**e**_ ∈ {1, 2, 4, 8, 16, 32, 64, 128, 256, 512} and the results are presented in Table 4. From the numbers, we can conclude that partitions resulting from other noise groups are as similar as within the same noise group for most levels of terms used. However, we again see that for the small vocabulary groups (induced with a high level of noise) there seems to be more discrepancy, especially when 3 terms are used, which might help to further explain the lower performance in previous experiments on those groups.

**Table 4:** Estimating the secondary effects of environmental noise, i.e., other than the number of terms used. For each number of terms used (column one) the table shows the internal consistency, measured using adjusted Rand index, for all generated maps (column two), and the mean internal consistencies computed for each noise level (column three), e.g. if the maps that ended up using 6 terms all where generated using 100 and 200 ***ϵ***_**e**_ noise then this column would be the average of the internal consistency of those two groups. 95% confidence interval indicated within parenthesis.

Terms used	All	Within noise group
3	0.273(±0.034)	0.324(±0.039)
4	0.337(±0.028)	0.377(±0.069)
5	0.373(±0.023)	0.275(±0.021)
6	0.537(±0.033)	0.486(±0.099)
7	0.593(±0.028)	0.632(±0.095)
8	0.518(±0.017)	0.573(±0.146)
9	0.510(±0.007)	0.541(±0.048)
10	0.549(±0.008)	0.521(±0.178)
11	0.543(±0.009)	0.538(±0.096)

### Modulating the vocabulary size by varying communication noise

**Fig 14:**
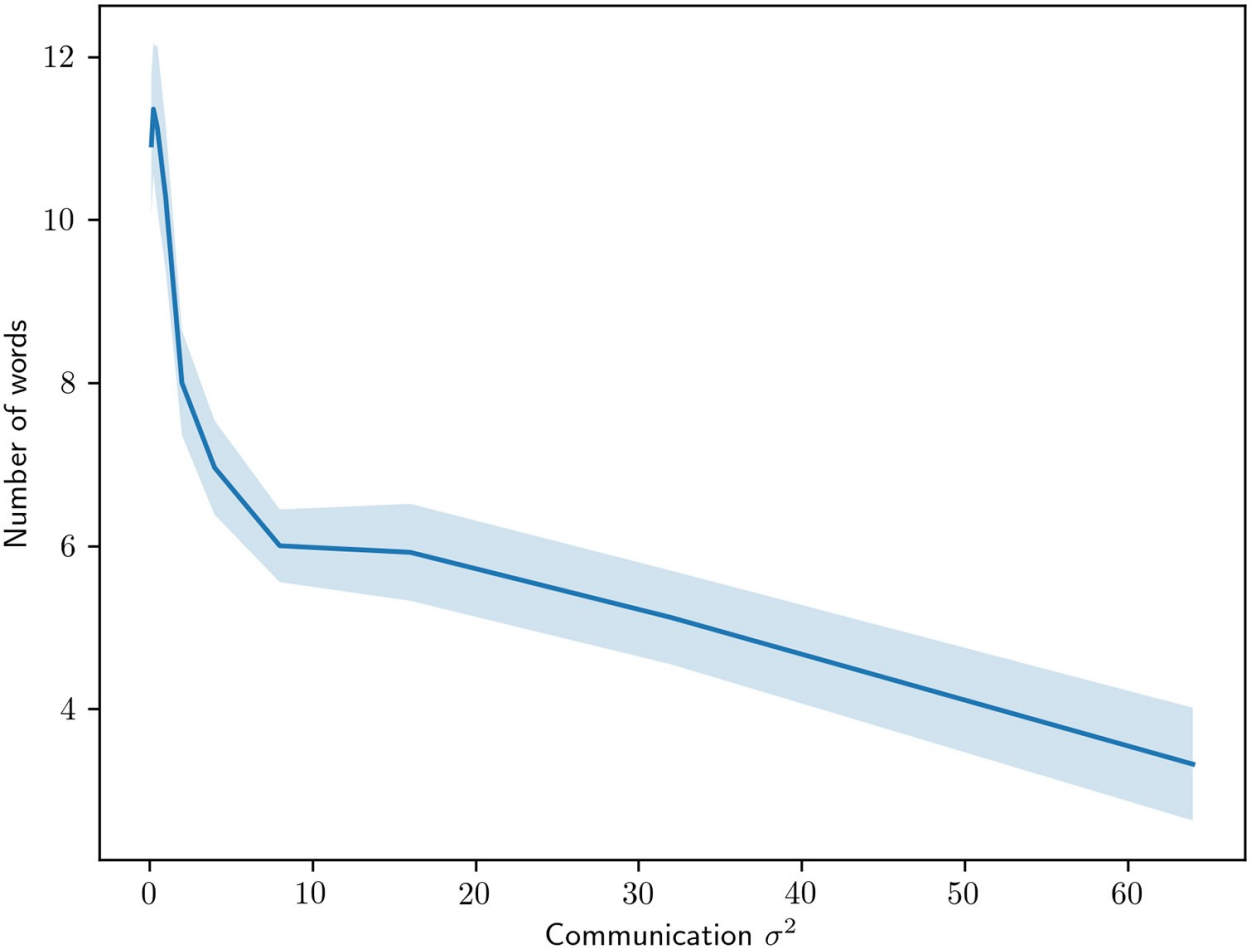
The number of color terms used by the agents when different amounts of noise are applied to their communication.

In order to investigate the effect of noise further, we turn to the noise on the communication channel over which words are transmitted. In Fig 14, we show how the number of words is affected when noise is introduced to the communication channel. In similarity with environmental noise, we see a decline in the number of terms used as we increase the noise in the communication channel. However, the characteristics seem to differ slightly where communication noise has a greater initial effect and then levels out.

## Materials and methods

### CIELAB correlation clustering

The CIELAB clustering is the partitioning obtained by applying correlation clustering [31, 32], a technique to obtain clusterings when there are both similarity and dissimilarity judgments on objects. This is applied to a graph with vertices corresponding to color tiles and where the edge (*u*, *v*) has weight $sim\left(u,v\right)-\frac{1}{2}$ where *sim* is the similarity metric defined in Eq (8). Thus, there are both positive weights corresponding to similar tiles (*sim* > 1/2) and negative weights corresponding to dissimilar tiles (*sim* < 1/2). Correlation clustering is a NP-hard problem, so we have used a new method that we developed based on a non-convex relaxation that is guaranteed to converge to a local optimum (forthcoming).

### Consensus maps by correlation clustering

In order to obtain a consensus maps of several different runs of our RL algorithm, we again use correlation clustering. Each run of our algorithm provides a similarity judgment between two tiles if they are placed in the same color partition and a dissimilarity judgment otherwise. We use these judgments as input to the correlation clustering algorithm to produce the consensus partition that aggregates all these judgments together.

### REINFORCE

REINFORCE [33] is a well known reinforcement learning algorithm in the policy gradient family, meaning that the policy is directly parameterized by a differentiable model, in our case a neural network. The model is trained to maximize expected reward by updating the neural network that suggests what actions to take to increase the probability of actions that have previously led to rewards in similar situations.

### Adjusted Rand Index

The Adjusted Rand index [37] is a method of computing the similarity between two data clusterings or partitions that was introduced by William M. Rand. Essentially it computes the relative number of pairs of objects that appear together in the same class in both partitions.

### The World Color Survey

The World Color Survey (WCS) [38] is a project that compiled color naming data from 110 unwritten languages and made it publicly available at http://www1.icsi.berkeley.edu/wcs/data.html. For each language, an average of 25 speakers were asked to name each color in a matrix of 330 color chips (see Fig 9) sampled from the Munsell color system to uniformly cover the human visual color spectra.

## Discussion

We see in Figs 6 and 7 that the RL results track the human results very closely in the KL loss and well-formedness characteristics. In terms of Rand index similarity (Table 2), the overall similarity of human languages to other human languages and RL mode maps to other RL mode maps is much greater than human language to RL mode maps at each number of words used. The human-to-RL similarity, however, is consistently greater than the human-to-random similarity, which is zero. Taken together, the reinforcement learning process produces mode maps that take into account some factor of human color space partitioning, and it also produces well-formedness and efficiency outcomes that represent a model significantly closer to the human behavior relative to these latter criteria.

One explanation for this difference may be found by looking at the Rand index similarity of the human-generated mode maps to the CIELAB maps. The latter is an idealized partitioning of the space based on color distances taken from CIELAB’s perceptually uniform (relative to human vision) color space. The similarity is consistently, but not hugely greater between the human maps and CIELAB than between the human maps and the RL maps. Given the success of the RL maps at modeling the communication characteristics of human color maps, this difference likely reflects biological and environmental aspects of human color perception that the simulated agents, due to their simplicity, do not represent. The RL-based maps also show similarly high Rand index similarity to the CIELAB maps, possibly due to the influence of the CIELAB distances on the reward function in the RL process. Our RL model therefore successfully separates communicative factors from the details of human perception, and gives space for experimentation on the influence of biological and environmental detail in arriving at a color term consensus within a simulated speech community.

Looking at the color maps in Fig 9, we perceive qualitatively *some* similarity in overall partitioning between humans and RL agents for a given number of color words, but the RL agents still do not closely replicate the human partitions—unsurprising, given the Rand index differences as above. The principal difficulty that the RL agents seem to have is in replicating human light/dark distinctions, which are under-emphasized in the RL partitions. We hypothesize that the light/dark distinction needs a different treatment, for reasons posed by the human perceptual architecture (for example, non-uniform need probabilities [2]), than the other components of the CIELAB or WCS color spaces.

On the other hand, the RL maps do share the behavior of the human maps with regard to how partitions of the color space are refined as we increase the number of colors used: the resulting partition tends to constitute a sub–partition rather than producing a completely different partitioning. Thus, the RL results appear to confirm the behavior observed by Berlin and Kay [24].

As argued in [3], there are trade-offs between cognitive and communication costs which could change over time in response to various evolutionary forces. Such changes may be quite difficult to study in real languages, but our framework provides a very powerful and flexible tool for studying such changes under carefully controlled conditions where we can adjust one parameter (say noise) while keeping the rest fixed.

## Conclusion

In this work, we successfully demonstrated the value of a reinforcement learning approach to simulating the conditions under which speakers might come to an agreement on how to partition a semantic space. Color provided a convenient domain of experiment because of the extent of real-world data collection and analysis that has already been performed and also due to the ability to represent the color space as evenly-selected samples from a continuous space, as with the WCS. Our RL agents replicate important aspects of human color communication, even though they lack the full perceptual and linguistic architecture of human language users. However, the RL paradigm will enable us in future work to represent more detailed aspects of the environment and biological architecture *in silico*, allowing our system to be used as a platform for hypothesis generation and cognitive modeling.

As for hypothesis generation, the behavior of our model suggests that greater communication and environmental noise produces an overall drop in the number of color words. This outcome provides further clues as to where to look for environmental factors that may account for differences in color vocabulary across real-world speaker groups.

Our approach can offer complementary insight to the recent approach of [22] who argued that languages efficiently compress ideas into words by optimizing the *information bottleneck*. Additional future work includes expanding from a two-agent paradigm to a multi-agent and even a large-population paradigm, which are areas under active development in the field of agent simulation. A key long-term goal for this work is to expand from the domain of color to other semantic domains, such as culture-specific partitions of approximate number (e.g., “few” vs. “many”) and even “general-domain” semantic relatedness hierarchies, such as WordNet.
